# Gravity evidence for a heterogeneous crust of Mercury

**DOI:** 10.1038/s41598-023-46081-4

**Published:** 2023-11-13

**Authors:** Salvatore Buoninfante, Maurizio Milano, Barbara Negri, Christina Plainaki, Giuseppe Sindoni, Maurizio Fedi

**Affiliations:** 1 Department of Earth, Environment and Resources Sciences, Università degli Studi di Napoli Federico II, Naples, Italy; 2 Istituto di Astrofisica e Planetologia Spaziali (IAPS), INAF, Rome, Italy; 3 Agenzia Spaziale Italiana (ASI), Rome, Italy

**Keywords:** Inner planets, Geophysics

## Abstract

We modeled gravity data to explore Mercury’s internal structure and show the presence of crustal heterogeneities in density. We first evaluated the lithospheric flexure occurring in the spherical harmonic degree range 5–80, according to the flexural isostatic response curve. We thus estimated a mean elastic lithosphere thickness of about 30 $\pm$ 10 km and modeled the crust-mantle interface, which varies from 19 to 42 km depth, according to a flexural compensation model. The isostatic gravity anomalies were then obtained as the residual field with respect to the contributions from topography and lithospheric flexure. Isostatic anomalies are mainly related to density variations in the crust: gravity highs mostly correspond to large-impact basins suggesting intra-crustal magmatic intrusions as the main origin of these anomalies. Isostatic gravity lows prevail, instead, above intercrater plains and may represent the signature of a heavily fractured crust.

## Introduction

The study of the internal structure of Mercury is fundamental for understanding the formation and evolution of the planet and of the entire Solar System. Mercury is constituted by a very large iron-rich core with a radius of ~ 2000 km, a ~ 400 km thick mantle made of iron-poor and magnesium-rich silicates, and a silicate crust with an average thickness of ~ 35 km. The core is divided into a solid inner core with a radius between 1000 and 1500 km, while the outer core is made up of convecting liquid of iron-sulfide, which is also responsible for the magnetic field. The exploration of Mercury began in 1974, when the NASA Mariner 10 (M10) spacecraft performed the planet’s first flyby on March 24. Thirty years later, NASA planned the MESSENGER mission, launched on August 3, 2004. The spacecraft performed one flyby of Earth (August 2005), two flybys of Venus (October 2006 and June 2007), and three flybys of Mercury (January 2008, October 2008, and September 2009, respectively). MESSENGER entered the orbit of Mercury on March 18, 2011^1,2^. The MESSENGER mission provided a large amount of data that allowed an in-depth study of Mercury’s density, magnetic susceptibility, composition, tectonic structure, volcanology, internal structure, exosphere, and magnetosphere^3^. Nowadays, the exploration of Mercury continues with the ESA-JAXA BepiColombo mission.

Thanks to MESSENGER, we obtained fundamental information on the planet’s gravity field, geological structure, and history. In particular, the MESSENGER Mercury Dual Imaging System (MDIS) provided images of the surface of the planet, allowing the recognition of geological units and structures. The Mercury Laser Altimeter (MLA) returned the surface topography (see, for instance, Manheim et al.^4^ and references therein), while Radio Science (RS) provided gravity field data by the spacecraft position and velocity.

Smith et al.^5^ computed the first global gravity field model HgM002, using the radio tracking data acquired by MESSENGER up to August 23rd, 2011. Despite the limited spectral resolution, up to spherical harmonic (SH) degree and order 20, this model allowed estimating the crustal thickness, the elastic lithosphere thickness of the Northern Rise region and the polar moment of inertia^5^. Later, Mazarico et al.^6^ and Mazarico et al.^7^ improved the gravity field models, up to SH degree and order 50 (HgM005) and 100 (HgM007), respectively. It provides a better relationship with the surface topography resulting from the measurements of MESSENGER's Mercury Laser Altimeter^8^. Genova et al.^9^ elaborated the HgM008 model, which defines the gravity field up to spherical harmonic degree and order 100. The latest gravity field models^10,11^, respectively named MESS160A and HgM009, are defined up to SH degree and order 160, corresponding to $\sim$ 50 km spatial resolution. Previous works provided maps of the free-air and Bouguer gravity anomalies (e.g., Smith et al.^5^; Mazarico et al.^6^; Genova et al.^9^; Konopliv et al.^10^; Genova et al.^11^; Verma et al.^12^). Bouguer anomalies are given by the difference between free-air anomalies and the gravity effect of topography, or Bouguer correction (see “Methods”).

The main purpose of this work is to analyze the MESS160A gravity field to investigate two possible crust models on a global scale. In fact, gravity data can be modeled by: (a) assuming a homogeneous crust, that is neglecting intra-crustal sources, or (b) assuming a crust that is possibly heterogeneous in density. In the first case, both spectral and inversion methods are used to estimate the crustal and elastic thickness. Other approaches use the dual inversion of gravity and topography^13^ or of dynamic pressure and crustal thickness^14,15^.

Wieczorek and Phillips^16^ suggested to model the crust-mantle interface by first performing a regularized downward continuation of the Bouguer anomalies down to an average depth and then converting it into the relief along the crust-mantle interface using an iterative approach^16,17^. The method was used to construct a global crustal thickness map for Mercury^9,14,18^. However, instabilities due to downward continuation occur, especially for continuations greater than the data sampling step (e.g., Wieczorek^17^; Bouman^19^; Fedi and Florio^20^). Under the approach (b) we also encounter an inherent instability, occurring when the continuation is close to the top of intra-crustal sources. In fact, if the crust is assumed not to be homogeneous (approach b) the anomalous field of Bouguer gravity can no longer be continued downwards to the crust-mantle interface, because Laplace equation applies (e.g., Blakely^21^; Fedi and Florio^20^) only in the harmonic region, that is in a source-free volume. Even though the problem may be somewhat regularized by low-pass filtering^16,22^, we will use in this paper a different method, which is more consistent with the approach (b).

Regarding the models of crustal thickness, it was estimated in a range of 0–90 km by James et al.^13^ and 0–70 km by Phillips et al.^18^. Watters et al.^14^ estimated two different crustal thickness models with depths ranging $\sim$ 11–73 km and ∼7–74 km, respectively.

Other authors estimated the mean crustal and elastic thicknesses of Mercury, or computed them in limited areas, by a spectral approach. Their method is based on the calculation of the admittance or correlation functions between gravity and topography (see Wieczorek^17^). Padovan et al.^23^ assumed the Airy isostatic model for 9 < $l$<15 degrees and derived a mean crustal thickness of 35 $\pm$ 18 km for the Mercury’s northern hemisphere using geoid-topography ratios; Sori^24^ calculated a mean crustal thickness of 26 $\pm$ 11 km, again using the Airy model. Konopliv et al.^10^ computed a crustal thickness ranging 23–50 km (see Table 1).

**Table 1:** Global crustal parameters estimated in previous works.

Elastic thickness, *T*_*e*_		Crustal thickness models		Mean crustal thickness, *T*_*c*_	
Value	Reference	Value	Reference	Value	Reference
31 $\pm$ 9 km	James et al.^25^	0–90 km	James et al.^13^	35 $\pm$ 18 km	Padovan et al.^23^
40 km	Watters et al.^26^	0–70 km	Phillips et al.^18^	26 $\pm$ 11 km	Sori^24^
25–30 km	Nimmo & Watters^27^	$\sim$ 11–73 km	Watters et al.^14^	23–50 km	Konopliv et al.^10^
		∼7–74 km	Watters et al.^14^		
$\sim$ 110–180 km	Tosi et al.^28^	8–97 km	Beuthe et al.^29^		

Regarding the mean elastic thickness (${T}_{e}$) of Mercury, James et al.^25^ estimated ${T}_{e}$ = 31 $\pm$ 9 km; Watters et al.^26^ derived ${T}_{e}$ = 40 km from the maximum depth of faulting; Nimmo and Watters^27^ calculated ${T}_{e}$= 25–30 km equating the elastic–plastic bending moment to the elastic bending moment. Very differently values, ${T}_{e}\cong$ 110–180 km, were estimated by Tosi et al.^28^, for degrees $l$= [2,4] of geoid and topography (see also Table 1). Differently from the above methods, some authors considered also crustal heterogeneities, in areas where surface and internal loads can be assumed to be in phase, i.e., when the two are related linearly by a degree-independent constant (e.g., Wieczorek^17^; Grott and Wieczorek^30^; Broquet and Wieczorek^31^). For these restricted areas, Goossens et al.^32^ estimated a crustal thickness ranging between 36 $\pm$ 14 and 112 $\pm$ 17 km and an elastic thickness between 11 $\pm$ 7 and 28 $\pm$ 7 km, while Genova et al.^11^ estimated crustal thickness varying between 51 $\pm$ 37 and 91 $\pm$ 22 km, and an elastic thickness varying between 5 $\pm$ 4 and 102 $\pm$ 35 km (see also Table 1). Beuthe et al.^29^ utilized the crustal densities derived from XRS data^33^ and obtained an 8–97 km crustal thickness model. In their model, crustal heterogeneity vs. depth is modelled by an exponential decrease of porosity and a smoothing out of lateral density variations.

We also consider a heterogenous model of the crust but employing a different procedure. To isolate the anomalies related to possible intra-crustal contributions, we model the lithospheric flexure regardless of the gravity field. We then get isostatic anomalies by subtracting to Bouguer anomalies the gravity effect caused by the lithospheric flexure.

## Results

We here describe our method to identify the contribution of possible intra-crustal heterogeneities to the Mercury’s gravity field. We first estimate the mean elastic thickness of Mercury, which will contribute to determine the effect of the crustal isostatic roots (isostatic gravity effect), compensating for the mass of the topographic reliefs. Finally, we will present the map of isostatic anomalies of Mercury, which may be interpreted as caused by sources of mainly crustal origin.

### Elastic thickness ${T}_{e}$ estimation

The estimation of the elastic thickness is essential to understand the elastic behavior of the lithosphere and to model the isostatic compensation surface.

**Figure 1:**
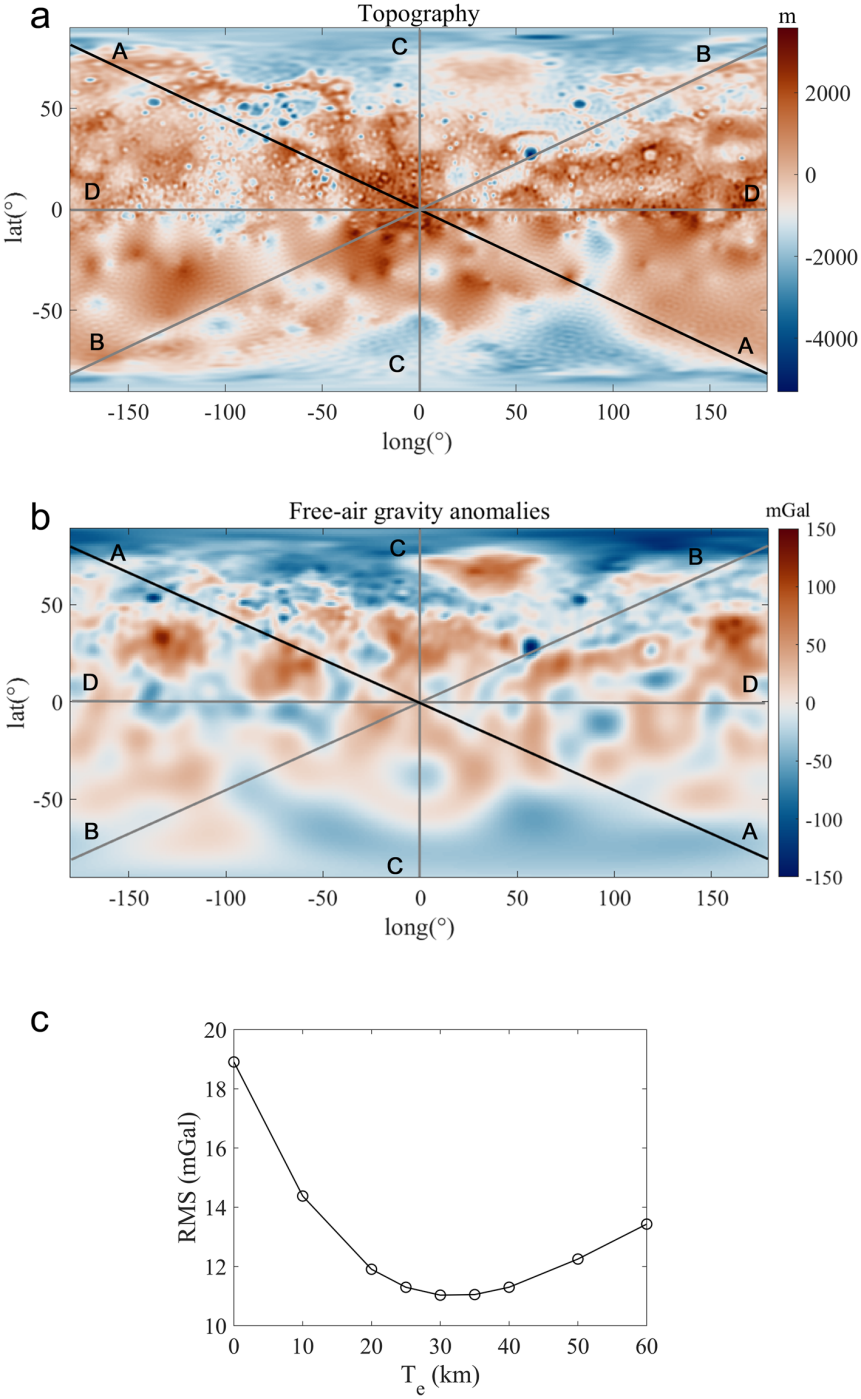
(**a**) Global topography of Mercury derived from the *GTMES_150v05* model in equirectangular projection. The black line indicates the representative global profile (*A*) among others (in gray) chosen to calculate the average elastic thickness; (**b**) Free-air gravity anomalies derived from the spherical harmonic model MESS160A in equirectangular projection. In Figs. 1a and b we used the Vik^35^ colormap to prevent ambiguity and visual distortion of the data; (**c**) Plot of the root mean square difference (RMS) between observed and calculated free-air gravity anomalies vs ${T}_{e}$, for the representative profile (*A*). RMS plots of the other chosen profiles are shown in Supplementary Fig. 2.

Following Watts^34^, we may search for the average elastic lithosphere thickness as the one giving the best fit between observed and calculated free-air anomalies along a series of 1-dimensional profiles. In Fig. 1 we show the results obtained along a NW–SE profile (Fig. 1a). The L_2_ misfit between the observed and calculated data (Fig. 1c) reaches its minimum at the elastic thickness of 30 km (see also Supplementary Fig. 3). By also evaluating the results obtained for other chosen profiles, we may assume ${T}_{e}$= 30 $\pm$ 10 km as representative of the mean elastic lithosphere thickness. We note that it is consistent with previous estimates (e.g., James et al.^25^; Nimmo and Watters^27^; Goossens et al.^32^).

### Airy or flexural compensation model?

**Figure 2:**
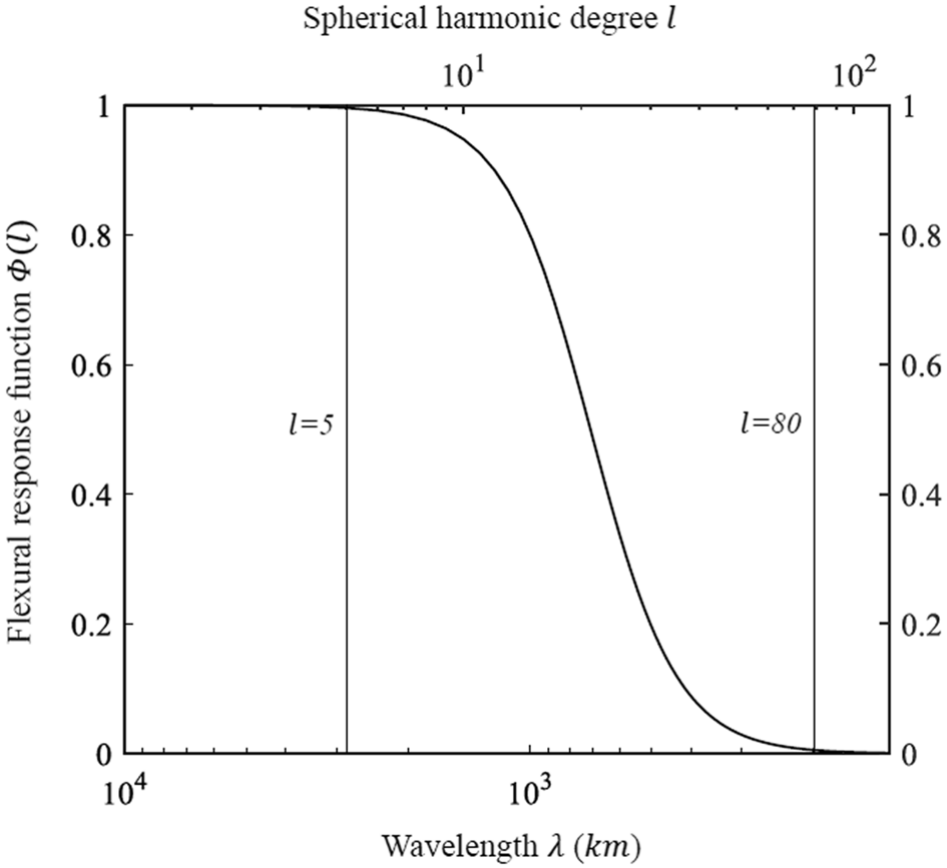
Flexural response function $\Phi \left(l\right)$ as a function of spherical harmonic degree $l$ and the effective Cartesian wavelength $\lambda =\frac{2\pi R}{\sqrt{l(l+1)}}$^36^, for an elastic thickness ${T}_{e}=$ 30 km. For degrees $l$<5, the model approximates Airy compensation $(D\to 0)$. For degrees $l$>80 topography is essentially uncompensated, approaching the Bouguer infinite rigidity case ($D\to \infty$). Flexural isostasy prevails in the spherical harmonic degree range 5 < $l$<80. The range of spherical harmonic degree 5 < $l$<80 corresponds to the range of wavelengths 190 < $\lambda$<2800 $km$.

The flexural response function (see “Methods”) represents the flexural response of the lithosphere to loading and allows understanding what type of isostatic model should be considered for a given spherical harmonic degree range. By analyzing the flexural response (Fig. 2), it is possible to determine the wavelength range corresponding that approximates an Airy compensation model (very low flexural rigidity), to the flexural compensation model (flexural rigidity), or to a non-compensation (very high flexural rigidity). The flexural response curve in Fig. 2 shows that the lithosphere of Mercury flexes for spherical harmonic degrees of the topography in the range 5 < $l$<80, i.e., or topography wavelengths ranging 190 < $\lambda$<2800 km. For spherical harmonics up to degree 5 and wavelengths longer than 2800 km, the flexural rigidity $D\to 0$, and we may assume the Airy compensation model. For surface features with wavelengths shorter than 190 km (l > 80), $D\to \infty$ so that no lithospheric flexure occurs since the rigidity of the lithosphere is such to resist the topographic load.

While the Airy compensation model is valid for $l$ < 5, components up to $l$ = 4 are associated with the polar mass deficit and to the morphological contrast between the lowlands and elevated regions^37^. Hence, we decided to subtract the terms up to degree 4.

### Isostatic gravity anomalies

**Figure 3:**
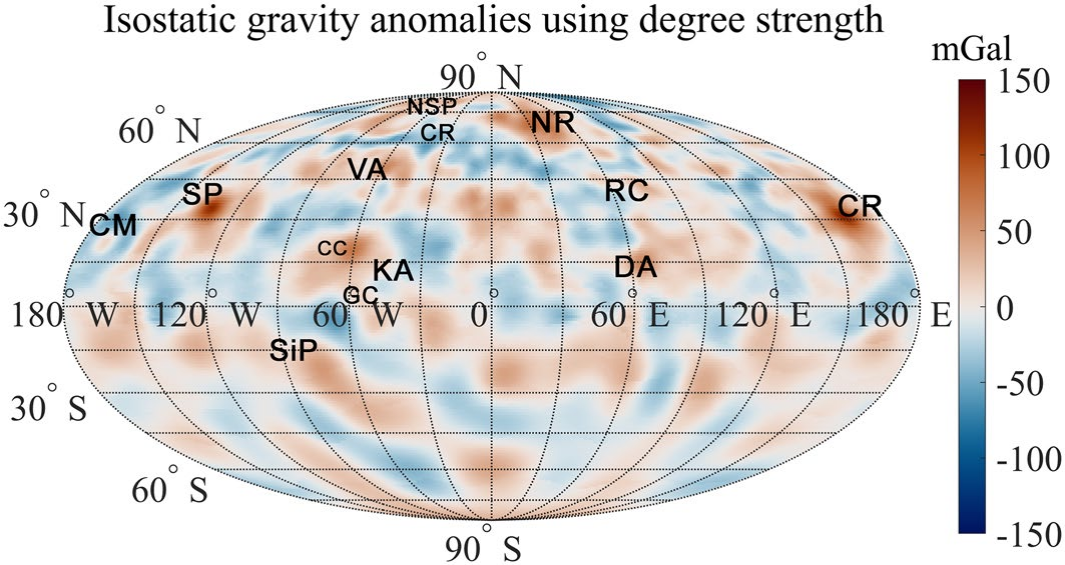
Isostatic gravity anomalies, after correcting the free-air anomalies with the topographic and isostatic gravity effects. For each point, the gravity is evaluated up to a maximum spherical harmonic degree that corresponds to the degree strength of the model MESS160A. Major features indicated are: **CM** Caloris Montes, **CP** Caloris Planitia,**CR** Carneige Rupes,**DA** Derain anomaly, **KA** Kuiper anomaly, **NR** Northern Rise, **NSP** Northern Smooth Plains, **RC** Rachmaninoff Crater, **SiP** Sihtu Planitia, **SP** Sobkou Planitia, **VA** Victoria Anomaly, **CC** Catullus Crater, **GC** Giotto Crater. The anomalies are computed at 50 km altitude (see Supplementary Information). Maps are in Mollweide projection.

The gravity effect of the topography may be evaluated by Eq. (4) in “Methods”. This equation can also be used to compute the isostatic gravity effect, by replacing the ${h}_{lm},{k}_{lm}$ coefficients of the topography in Eq. (5) with the lithospheric deflection coefficients of the crust-mantle interface ${w}_{lm},{u}_{lm}$ (see “Methods”). The map of the isostatic gravity, i.e., the isostatic correction assuming a flexural model, is shown in Supplementary Fig. 6. In Fig. 3 we show the isostatic gravity anomalies (from now on we refer to isostatic gravity anomalies as Bouguer anomalies corrected for the gravity effect of compensation assuming a flexural model), obtained by subtracting the topographic and the isostatic gravity effects from the free-air anomalies and constrained by the MESS160A degree strength map (Fig. 4 in Konopliv et al.^10^), which shows the local resolution of the gravity field, i.e., the maximum acceptable degree of the gravity field model for given latitudes and longitudes. It is derived at the intersection between the expected acceleration at the Mercury surface and the surface acceleration uncertainty^10,38^. Supplementary Figure 7 shows the map of isostatic anomalies without constraints on the degree strength. As expected, in Fig. 3 we note that the isostatic gravity anomalies get smoother where the degree strength is lower, compared to the anomalies calculated without degree strength constrain.

**Figure 4:**
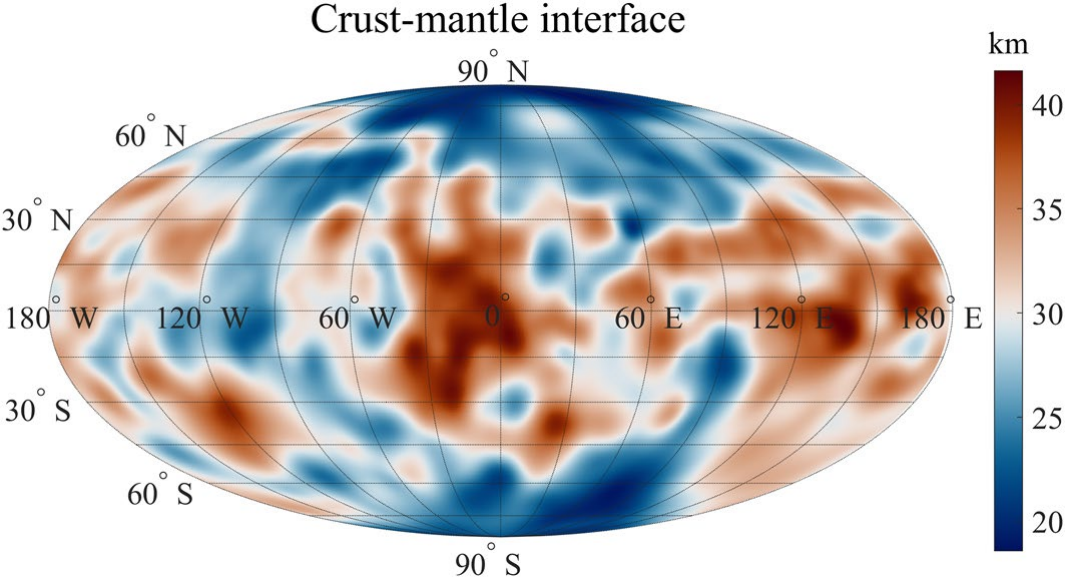
Map of the crust-mantle interface of Mercury predicted from a lithospheric flexure model, expressed as depth (km) with respect to the reference surface, in a Mollweide projection. We modeled the deflection of the crust-mantle interface using the estimated elastic lithosphere thickness of 30 km, the crustal thickness (*T*_*c*_) of 35 km and the densities of 2800 kg/m^3^ and 3200 kg/m^3^, for crust and mantle respectively.

### Crust-mantle interface modeling

We calculated lithospheric deflection coefficients (see “Methods”) to model the crust-mantle interface (Fig. 4) by means of a spherical harmonic expansion. We considered the previously estimated mean elastic thickness of 30 km and assumed densities for crust and mantle of 2800 kg/m^3^ and 3200 kg/m^3^, respectively^9^.

We neglected compensating roots of sub-surface loads, since the intra-crustal sources (intrusions, dikes, sills) are bodies with limited lateral extent. Indeed, the flexural response function $\Phi \left(l\right)$ (Supplementary Fig. 10) shows that, for intra-crustal sources at $\sim$ 15 km depth with lateral extension $\lesssim$ 500 km, and $\sim$ 300 kg/m^3^ density contrast, the lithospheric flexure tends to be negligible and does not generate any compensation roots effect. By a visual inspection of the extent of the isostatic anomalies in Fig. 3, which roughly accounts for the maximum size of the related source, sub-surface loads would thus hardly involve a crustal deepening that could affect the crust-mantle interface.

Our result shows that the crust-mantle interface has a depth variable between 19 and 42 km. This estimate is included in a narrower range of values than the previous estimates^9,13,14,18,29^, as the lithospheric rigidity effect has a strong influence on the crustal thickness modeling.

In order to assess the significance of our estimation of the crust-mantle interface depth, we performed a sensitivity analysis of that interface vs. topography model uncertainty. First, we computed the average value of the coefficient uncertainties of the topography model, which was estimated as $\pm$ 9.5%. Based on this, we then computed the sensitivity error of the depth to crust-mantle interface (Eq. (11) in “Methods”), yielding a 5% error.

## Discussion

The identification of isostatic gravity anomalies is crucial for better understanding Mercury’s internal structure. We may distinguish among isostatic highs and lows. Isostatic anomaly highs are mainly identified above large basins, which originated after huge meteoritic impacts and are mostly characterized by smooth plains on the surface^39–41^. These anomalies can mainly represent the effect of intra-crustal magmatic intrusions^42^ caused by the regional extensional regime following the impact, as also observed for the Moon and Mars^43^. On the other hand, isostatic anomaly lows are mainly found above intercrater plains regions, also named heavily cratered terrains^41^. These regions are likely characterized by a highly fractured crust with respect to the impact basin areas and possess a lower crustal bulk density. In principle, isostatic anomaly lows could also be explained by the presence of voids, interpreted as empty lava tubes or caves, as identified on Earth, Moon and Mars^44^ (e.g., Schröter’s Rille^45^, Marius Hills Hole^46^). However, these effects are expected not to be important at the scale we are discussing. We stress that a significant part of the anomalies in our maps was not evident in the free-air and Bouguer anomaly maps, because of the interference with the effect of the topography and of the crust-mantle interface. Above the Northern Smooth Plains (**NSP** in Fig. 3), many anomalies characterized by wavelengths of a few hundred km and clearly visible in the isostatic anomaly map, are difficult or not at all detectable in the free-air and in the Bouguer anomaly maps (Supplementary Fig. 1a and d, respectively). Similarly, in the southern polar area the isostatic anomaly map highlights a gravity high extending in latitude from 57°S to 90°S, and flanked towards the west by a low, which corresponds to a depressed region of the southern hemisphere. An extended free-air gravity low is, instead, observed in the same area, while the Bouguer anomaly map is dominated by anomaly highs. In the Rachmaninoff crater (**RC**) area, within which lies the lowest elevation on Mercury^47^, the free-air anomaly map shows an intense low. The Bouguer anomaly map shows a high, probably due to the mantle uplift following the formation of the crater^48^. On the other hand, the isostatic anomaly map in Fig. 3 does not present significant anomalies, thus implying that scarce magmatic intrusion phenomena occurred. The isostatic anomaly map shows several anomalies associated in the topographically highest area of Mercury, between latitude 45°S-35°N and longitude 300°E–30°E. Here the Bouguer anomaly map shows an intense low, which does not allow us to identify and interpret other shorter wavelength anomalies. On the contrary, the free-air anomaly map is characterized by overlapping anomalies which hide the effects associated with intra-crustal sources.

Previous studies interpreted the Bouguer gravity anomaly in the Caloris basin (**CP**) and the Sobkou basin (**SP**) areas, as associated with topographically lows and mass concentrations (mascons)^6,10^; however, the presence of isostatic anomaly highs in the same areas seem suggesting an interpretation in terms of intra-crustal magmatic intrusions.

For the Northern Rise (**NR**) anomaly, two main hypotheses have been made about its origin. The first hypothesis considers a crustal or even deeper source related to the mantle uplift, following a meteoritic impact^8^. The latter considers a source in the core or at core-mantle boundary interface^49^. Once again, the concurrent presence of an isostatic anomaly seems suggesting the occurrence of possible magmatic intrusions.

The isostatic anomaly high named Victoria anomaly (**VA**) is found in the more depressed area of the Victoria Quadrangle (301°E, 49°N), which is associated with an impact basin (b55^45^ in Supplementary Figs. 8, 9). As indicated in Supplementary Fig. 8, isostatic anomaly highs are frequently associated to impact basins and, therefore, can be interpreted by possible intrusive bodies.

Other anomaly highs such as the anomaly observed in the region between the Catullus (**CC**) and Giotto (**GC**) craters, in the Kuiper Quadrangle (**KA**), and the anomaly south of the Rachmaninoff crater (**CR**), the Derain anomaly (**DA**) (see Fig. 3), do not show any correlations with the topography and seem not associated with any known impact basin, so leading to a more difficult geological interpretation.

Eastward the Caloris Basin (**CP**) anomaly, the isostatic anomaly map shows an alignment of gravity lows, corresponding to the Caloris Montes (**CM**). In the northern polar region, we see a large isostatic anomaly low, extending from 77°N to 90°N latitude, and from 110°E to 200°E longitude, which corresponds to geological units known as ‘smooth plains’. Finally, an alignment of isostatic anomaly lows runs along the tectonic structure known as Carneige Rupes (**CR**) (250°E-325°E, 55°N-65°N), one of the largest lobate scarps in the northern hemisphere of Mercury, indicating the presence of crustal density discontinuities along this structure. The isostatic anomaly low west of Sihtu Planitia (**SiP**) (293°E, 5.5°S), included within the High Mg-Region, shows no correlation with the topography, thus suggesting that it is generated by an intra-crustal source.

Following Goossens et al.^32^, the elastic thickness is related to heat flux through the Eq. (7). Elastic thickness is related to time of topography formation during the LHB and to later resurfacing (e.g., Marchi et al.^50^, Byrne et al.^51^), in the period between 4.2 and 3.5 Ga. We assume that the temperature at the base of the lithosphere is 1050 K (e.g., Breuer & Moore^52^, Goossens et al.^32^), the surface temperature is 440 K (e.g., Padovan et al.^53^, Goossens et al.^32^) and the thermal conductivity is 3 $W {\mathrm{m}}^{-1}$ ${\mathrm{K}}^{-1}$ (e.g., Michel et al.^54^, Goossens et al.^32^). The resulting heat flux *q* for ${T}_{e}$= 30 km is 61 $\mathrm{mW }{\mathrm{m}}^{-2}$. Watters et al.^26^ and Nimmo & Watters^27^ found that the heat flux ranges between 10 and 50 $\mathrm{mW }{\mathrm{m}}^{-2}$. Later, Goossens et al.^32^ calculated a value of 105 $\mathrm{mW }{\mathrm{m}}^{-2}$ for the planet heat flux. Our results present relatively high values of heat flux, which may be however expected for Mercury, as a planet partially molten at the end of accretion and subsequently rapidly cooling.

The conclusions of our study may be summarized as follows:

1. The isostatic gravity anomalies provide a useful tool for the geological interpretation of Mercury;
2. The mean elastic lithosphere thickness calculated for Mercury is 30 $\pm$ 10 km;
3. The crust-mantle interface is 19 to 42 km deep. The larger crust-mantle interface depths (down to ~ 42 km) are reached in the equatorial regions, the smaller in the polar regions (down to ~ 19 km). The crustal roots thickening due to larger topographic loads is responsible for these depths;
4. Isostatic gravity highs are mostly interpreted with intra-crustal magmatic intrusions and isostatic gravity lows as the effect of a heavily fractured crust.

The isostatic gravity anomaly map provides a significantly useful tool for interpreting gravity data in terms of crustal sources and for modeling internal crustal structures. However, it must be considered that the resolution of gravity data is better in the Northern hemisphere as well as for topographic data, on which our calculations depend. Furthermore, these calculations depend on an estimated average elastic thickness globally and not for spherical caps. We hope that the current BepiColombo mission will provide a better data resolution on the southern hemisphere of Mercury. This will lead to an improved gravity field model and a more detailed isostatic gravity anomaly map, together with regional estimates of the elastic thickness.

## Methods

### Spherical harmonic expansion

The Newtonian gravitational potential *W* of a planet can be represented as the sum of a normal potential *U*, and a disturbing potential *T*. At a global scale, the gravity field is expressed through a series of spherical harmonics, which are the solutions of the Laplace equation in spherical coordinates (e.g., Barthelmes^55^):

1 $$W\left(r,\lambda ,\varphi \right)=\frac{GM}{R}\sum_{l=0}^{\infty }\sum_{m=0}^{l}{\left(\frac{R}{r}\right)}^{l+1}{P}_{lm}\left(\mathrm{sin}\varphi \right)\left[{C}_{lm}\mathrm{cos}m\lambda +{S}_{lm}\mathrm{sin}m\lambda \right],$$

where $\left(r,\lambda ,\varphi \right)$ are the geocentric coordinates of the satellite; $G$ is the gravitational constant; $M$ and $R$ are the mass and mean radius of the planet; ${C}_{lm}$, ${S}_{lm}$ are the fully normalized spherical harmonic coefficients; ${P}_{lm}$ are the fully normalized associated Legendre polynomials of degree $l$ and order $m$. The spherical harmonic coefficients (Stokes coefficients) are used to study the overall structure and the irregularities of the field in the spectral domain. The spherical harmonics expansion is truncated to a degree ${l}_{max}$, depending on the data resolution^13^.

The disturbing potential is defined as:

2 $$T\left(r,\lambda ,\varphi \right)=\frac{GM}{r}\sum_{l=2}^{{l}_{max}}{\left(\frac{R}{r}\right)}^{l}\sum_{m=0}^{l}{P}_{lm}\left(\mathrm{sin}\varphi \right)[{C}_{lm}^{T}\mathrm{cos}m\lambda +{S}_{lm}^{T}\mathrm{sin}m\lambda ].$$

A spherical approximation is often used for the disturbing potential $T$^56^; in addition, if the origin of the reference system coincides with the planet center of mass, the potential has no terms of degree $l$ = 1. Therefore, the sum of $T$ starts from $l$ = 2.

The free-air gravity anomaly ${g}_{FA}$ is given by^37,57^:

3 $${g}_{FA}\left(r,\lambda ,\varphi \right)= -\frac{\partial T}{\partial r}-\frac{2T}{r}=\frac{GM}{{r}^{2}}\sum_{l=2}^{{l}_{max}}{\left(\frac{R}{r}\right)}^{l}(l-1)\sum_{m=0}^{l}{P}_{lm}\left(\mathrm{sin}\varphi \right)\left[{C}_{lm}^{T}\mathrm{cos}m\lambda +{S}_{lm}^{T}\mathrm{sin}m\lambda \right].$$

### Topography gravity effect and Bouguer anomaly

We used *SHTools* software^58^ (https://shtools.oca.eu/shtools/public/) to estimate the gravity effect of the topography ${g}_{TE}$ as:

4 $${g}_{TE}\left(r,\lambda ,\varphi \right)=\frac{GM}{{r}^{2}}\sum_{l=0}^{{l}_{max}}{\left(\frac{R}{r}\right)}^{l}(l-1)\sum_{m=0}^{l}{P}_{lm}\left(\mathrm{sin}\varphi \right)\left[{H}_{lm}\mathrm{cos}m\lambda +{K}_{lm}\mathrm{sin}m\lambda \right],$$

with the topographic effect coefficients $\left\{{H}_{lm},{K}_{lm}\right\}$ defined as^16^:

5 $$\left\{{H}_{lm},{K}_{lm}\right\}=\frac{4\pi \Delta \rho {B}^{3}}{M(2l+1)} \sum_{n=1}^{l+3}\frac{\left\{{h}_{lm}^{n},{k}_{lm}^{n}\right\}}{{B}^{n}n!}\frac{\prod_{j=1}^{n}(l+4-j)}{(l+3)},$$

where $\left\{{h}_{lm},{k}_{lm}\right\}$ are the topography model coefficients, $B$ is the zero-degree term of the topography coefficients (i.e., the Mercury radius), $n$=5 the order of the Taylor series expansion. A $\Delta \rho$ = 2800 kg/m^3^ density is assumed for the underlying crust^9^. The Bouguer anomaly is given by the difference between the free-air anomaly and topographic gravity effect.

### Elastic thickness and heat flux

The isostatic gravity effect can be calculated assuming a local compensation model, i.e., a crust without rigidity that is effectively floating on a denser lower mantle^59,60^. However, we here assume a complex model, according to which the crust has an elastic behavior and the lithosphere flexes in response to surface loads^61,62^. According to the Vening Meinesz^61^ theory, the isostatic compensation occurs as a regional phenomenon, so that small topographic loads do not deform the lithosphere. The lithosphere acts as an elastic shell, whose strength distributes the topographic load over a wider horizontal distance. The greater the elastic thickness (${T}_{e}$), the greater the resistance of the lithosphere, and the lower the bending^63^. Following Turcotte et al.^62^, in this work we have however considered a model in which the plate strength is defined in terms of flexural rigidity or elastic thickness rather than radius of regionality. To estimate the elastic thickness, we used 1-dimensional profiles oriented in different directions. For each profile, and assuming different ${T}_{e}$ values, we compared the observed free-air gravity anomalies with the calculated free-air gravity anomalies, which are given by:

6 $${\text{FA}} = {\text{IE}} + {\text{TE}} + {\text{IA}},$$

where IE is the calculated isostatic gravity effect, TE the topography gravity effect and IA the isostatic gravity anomalies.

Assuming a linear temperature gradient, the heat flux *q* can be calculated using the Fourier’s law of heat conduction (e.g., Goossens et al.^32^; Turcotte & Schubert^64^):

7 $$q=k\frac{{{T}_{base}-T}_{surf}}{{T}_{e}}$$

where *k* is the coefficient of thermal conductivity, ${T}_{base}$ is the temperature at the base of the lithosphere and ${T}_{surf}$ the surface temperature.

### Lithospheric deflection coefficients

The lithospheric deflection and its gravity effect are dependent on the topography and on the crustal and mantle densities. Following Turcotte et al.^62^, a downward displacement *f* depresses the crust-mantle interface, resulting in an upward negative pressure which depends on the crust-mantle density contrast. It is thus implicitly assumed that the crust with density ${\rho }_{c}$ fills the flexured region^62^. Thus, the lithospheric deflection coefficients ${w}_{lm},{u}_{lm}$ can be obtained considering that the ratio between these coefficients and the topography coefficients ${h}_{lm},{k}_{lm}$ is:

8 $$\frac{{w}_{lm}}{{h}_{lm}}=\frac{{u}_{lm}}{{k}_{lm}}=\frac{{\rho }_{c}}{{\rho }_{m}-{\rho }_{c}}\left\{1-\frac{3{\rho }_{m}}{(2l+1)\overline{\rho }}\right\}{\left\{\frac{\sigma \left[{l}^{3}{\left(l+1\right)}^{3}-4{l}^{2}{\left(l+1\right)}^{2}\right]+\tau \left[l\left(l+1\right)-2\right]+l\left(l+1\right)-\left(1-v\right)}{l\left(l+1\right)-\left(1-v\right)}-\frac{3{\rho }_{m}}{\left(2l+1\right)\overline{\rho }}\right\}}^{-1},$$

where $\tau$ is the rigidity of the lithosphere if the bending resistance is neglected^62,65^, given by:

9 $$\tau =\frac{E{T}_{e} }{{R}^{2}g\left({\rho }_{m}-{\rho }_{c}\right)} ,$$

$\sigma$ the resistance of the lithosphere to bending:

10 $$\sigma =\frac{D}{{R}^{4}g\left({\rho }_{m}-{\rho }_{c}\right)}=\frac{\tau }{12\left(1-{v}^{2}\right)}{\left(\frac{{T}_{e} }{R}\right)}^{2},$$

where $D$ is the effective flexural rigidity and $\overline{\rho }$ the mean density (5429 $\frac{kg}{{m}^{3}}$)^10,66^.

### Sensitivity estimate

The sensitivity on the estimated crust-mantle interface depth is:

11 $$S=\frac{(CMI1-CMI2)}{CMI},$$

where $CMI$ is the estimated crust-mantle interface depth. $CMI1$ and $CMI2$ are the crust-mantle interface depths estimated by, respectively, incrementing and decrementing ${h}_{lm},{k}_{lm}$ of $\pm$ 9.5% of their value, i.e., by the average topographic coefficients error.

### Flexural response function

Following Watts^67^ the lithosphere can be considered as a filter between the load and the flexure, which suppresses the short-wavelength deformation and passes the long-wavelength deformation. The ratio between flexure and load is defined as flexural response function. This function is useful to understand where the lithosphere flexes, as a function of the applied topographic load. According to Watts and Moore^68^, the flexural compensation, or flexural response function $\Phi \left(l\right)$ (Fig. 2), depends on the spherical harmonic degrees of the topography. This function is given by:

12 $$\Phi \left(l\right)={\left[1+\frac{D}{\left({\rho }_{m}-{\rho }_{c}\right)g}{\left(\frac{2l+1}{2R}\right)}^{4}\right]}^{-1},$$

where ${\rho }_{c}$ is the crustal density, ${\rho }_{m}$ is the mantle density, $R$ is the planet radius and $D$ the effective flexural rigidity which is defined as:

13 $$D=\frac{E{T}_{e}^{3}}{12\left(1-{v}^{2}\right)},$$

where $E$ is the Young’s modulus, $v$ is the Poisson’s ratio, ${T}_{e}$ is the elastic thickness, $g$ is the gravitational acceleration.

When the flexural response function curve flattens and is $\sim$ 1, the flexural rigidity $D\to 0$, and we may assume the local compensation Airy model. If the flexural response function tends to zero, the flexural rigidity $D\to \infty$, and the topographic loads are considered too weak to cause an isostatic compensation. In the intermediate range, topographic loads cause a lithospheric flexure, and a regional compensation model (flexural model) can be assumed^68^. In Table 2 we provide the parameters used to calculate the flexural response function for Mercury.

**Table 2:** Parameter values used in this paper.

Parameter	Value	Reference
Crustal density, ρ_c_	2800 kg/m^3^	Genova et al.^9^
Mantle density, ρ_m_	3200 kg/m^3^	Genova et al.^9^
Planetary radius, *R*	2439.4 km	
Young’s modulus, *E*	100 GPa	Johnson et al.^65^; Dombard & Hauck^69^; Hauck et al^70^.
Poisson’s ratio, υ	0.25	Johnson et al.^65^; Hauck et al. ^70^; Klimczak et al.^71^
Elastic thickness, *T*_*e*_	30 km	
Crustal thickness, *T*_*c*_	35 km	
Gravitational acceleration, *g*	3.70 m/s^2^	

### Supplementary Information

Supplementary Information.

## Data Availability

MESS160A model coefficients can be downloaded from the NASA GSFC portal at https://pgda.gsfc.nasa.gov/products/71 or from the Geosciences Node of NASA Planetary Data System at http://pds-geosciences.wustl.edu/messenger/mess-h-rss_mla-5-sdp-v1/messrs_1001/. *GTMES_150v05* model coefficients can be downloaded at http://pds-geosciences.wustl.edu/messenger/mess-h-rss_mla-5-sdp-v1/messrs_1001/.
