# Supplementary material for: Gravity evidence for a heterogeneous crust of Mercury
Source: Sci Rep. 2023 Nov 13;13:19854. doi: 10.1038/s41598-023-46081-4 (PMC10646127; doi:10.1038/s41598-023-46081-4)
Supplement: Supplementary file 1 — Supplementary Information. [file 41598_2023_46081_MOESM1_ESM.docx]

Supplementary information for

**Gravity evidence for a heterogeneous crust of Mercury**

Salvatore Buoninfante^1,2^, Maurizio Milano^1*^, Barbara Negri^3^, Christina Plainaki^3^, Giuseppe Sindoni^3^, and Maurizio Fedi^1^

^1^ Department of Earth, Environment and Resources Sciences, University of Naples Federico II, Naples, (Italy), ^2^ Istituto di Astrofisica e Planetologia Spaziali (IAPS), INAF, Rome, Italy

^3^ Agenzia Spaziale Italiana (ASI), Rome, Italy

***** Corresponding author: Maurizio Milano [(maurizio.milano@unina.it)](mailto:(maurizio.milano@unina.it))

**Content of this file**

Free-air and Bouguer gravity anomalies

Airy isostatic gravity anomalies and spectral correlation

Supplementary Figures 1-10

**Free-air and Bouguer gravity anomalies of Mercury**

Supplementary Figure 1a shows the free-air gravity anomalies of Mercury calculated in this work using the MESS160A model coefficients (see Methods). We present here the gravity field maps at an altitude of 50 km, as it is representative of the last period (April 2014 - January 2015) of radioscience measurements of MESSENGER[^1^](#Genova)^,^[^2^](#Solomon). Supplementary Figure 1b shows the topography of Mercury calculated from the *GTMES_150v05* spherical harmonic model, defined up to degree and order 150[^3^](#Neumann). The model defines the shape of Mercury and is expressed in terms of radial distance from the center of mass. It is derived from the MESSENGER’s Mercury Laser Altimeter (MLA) measurements in the Northern hemisphere and from radio occultations measurements[^4^](#Perry) in the Southern hemisphere. The topography model *GTMES_150v05* has a resolution of the spherical harmonic degree similar to the resolution of the gravity field model. We may note that the free-air gravity anomalies correlate well with the topography, especially in the Northern hemisphere, where both have a good data resolution. In the Southern hemisphere the radioscience instrument acquired gravity data at high altitude, while the topography is derived by radio occultations observations only, as there are no altimeter data. Therefore, in this hemisphere the correlation between free-air gravity anomalies and topography is worse since the data resolution is lower. We estimated the Bouguer anomalies (Supplementary Figure 1d) by subtracting the topographic gravity effect to the free-air anomalies (see also Methods).

**Airy isostatic gravity anomalies and spectral correlation**

The Airy isostatic compensation model[^5^](#Airy) assumes that the lithosphere has no rigidity, and that, therefore, the elastic thickness $T_{e}$=0. Based on this model we also calculated the Airy isostatic anomalies, shown in Supplementary Figure 4, adapting the method proposed by Hirt et al.[^6^](#Hirt) for Mercury. We assumed the crustal thickness $T$=35 km, crust and mantle density $\rho_{c}$=2800 kg/m^3^ and $\rho_{m}$**=**3200 kg/m^3^, respectively[^1^](#Genova2019), and the mean density $\bar{\rho}$=5429 kg/m^3^ (see also <https://nssdc.gsfc.nasa.gov/planetary/factsheet/mercuryfact.html>). The map in Supplementary Figure 4 shows higher correlation to the free-air gravity map, with respect to the isostatic gravity map obtained assuming a flexural compensation model. This is clearly due to the excessive simplification imposed by the Airy model, which does not consider the effect of lithospheric rigidity and leads to an overestimation of the compensating crustal roots. The excessive correlation between free-air anomaly and Airy isostatic anomaly is also clear by analyzing their spectral contribution. The correlation between gravity and topography may be defined as a dimensionless quantity based on their cross-power spectra[^7^](#Wieczorek). Here, we replaced these cross-power spectra with the cross-power spectra of the free-air anomaly and isostatic anomaly fields:

$\gamma\left( l \right)=\frac{S_{fi}\left( l \right)}{\sqrt{S_{ff}(l)S_{ii}(l)}}$, (1)

where $S_{fi}\left( l \right)$ is the cross-power spectrum between the free-air anomaly and isostatic anomaly fields, $S_{ff}(l)$ is the power spectrum of the free-air anomaly fields and $S_{ii}(l)$ is the power spectrum of the isostatic anomaly fields.

We calculated and compared the spectral correlation $\gamma\left( l \right)$ between free-air and Airy isostatic anomalies and between free-air and flexural isostatic anomalies (Supplementary Figure 5). In both cases the correlation decreases as the spherical harmonic degree increases. However, the spectral correlation curve between free-air anomaly and flexural isostatic anomaly tends to zero over a much wider spherical harmonic degree range than the spectral correlation between free-air anomaly and Airy isostatic anomaly. This further confirms that a model considering lithospheric rigidity is necessary to isolate anomalies deriving from intra-crustal sources.


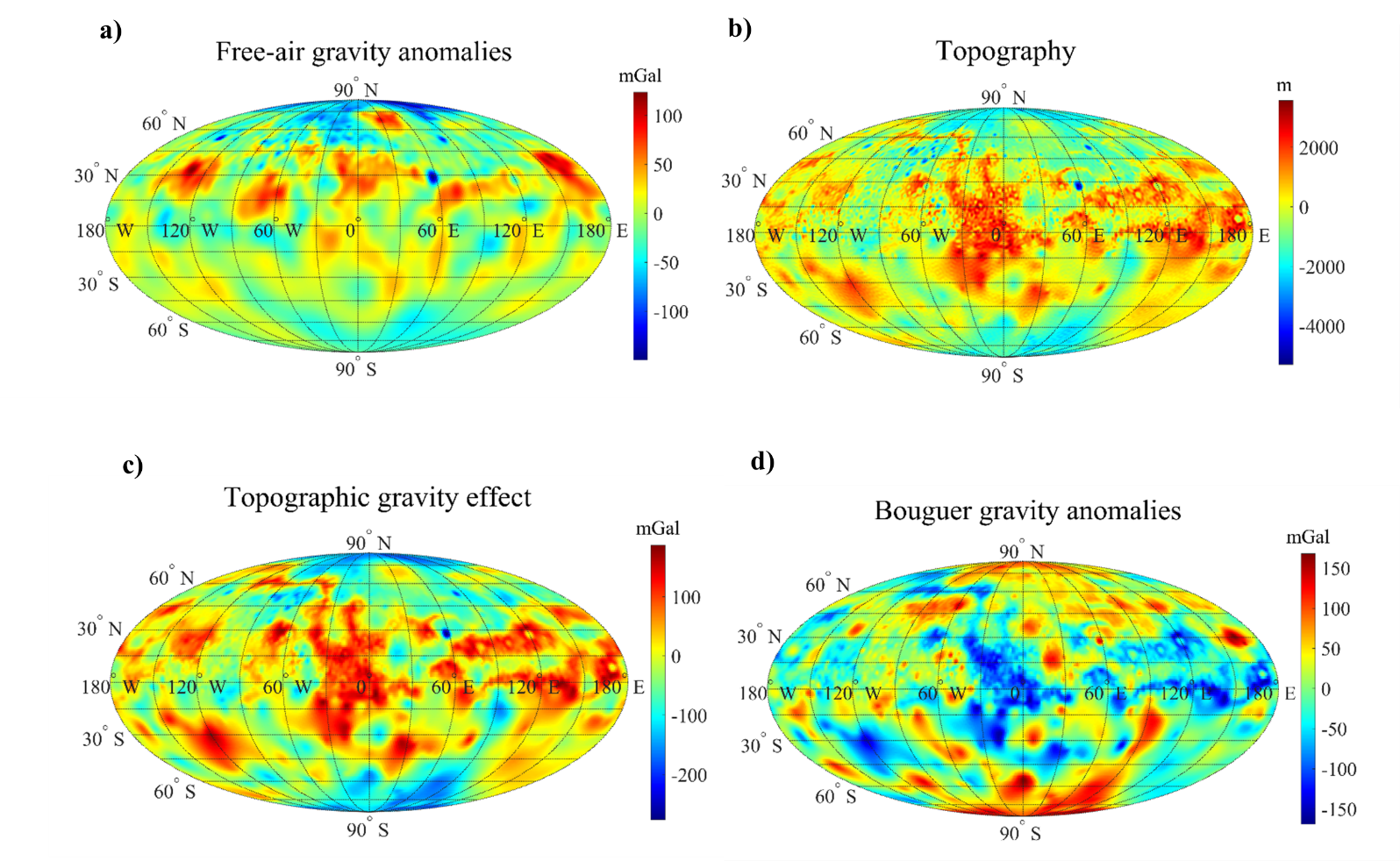
**Supplementary Figure 1. a**) Free-air gravity anomalies, from the spherical harmonic model MESS160A**; b)** Global topography of Mercury from the spherical harmonic model *GTMES_150v05*; **c)** Topographic gravity effect, from the spherical harmonic model *GTMES_150v05*; **d)** Bouguer gravity anomalies, from the spherical harmonic model MESS160A. The anomalies are computed at 50 km altitude. Maps are in Mollweide projection.

RMS between observed and calculated free-air anomalies

***Profile B***

***Profile C***

***Profile D***

**Supplementary Figure 2.** Root mean square difference (RMS) between observed and calculated free-air gravity anomalies vs $T_{e}$ for profiles *B,C* and *D*.

free-air anomalies

calculated free-air anomalies

Free-air gravity anomalies fitting

**Supplementary Figure 3.** Comparison between observed and calculated free-air gravity anomalies along a global profile NW-SE, for different elastic lithosphere thickness $T_{e}$.

**
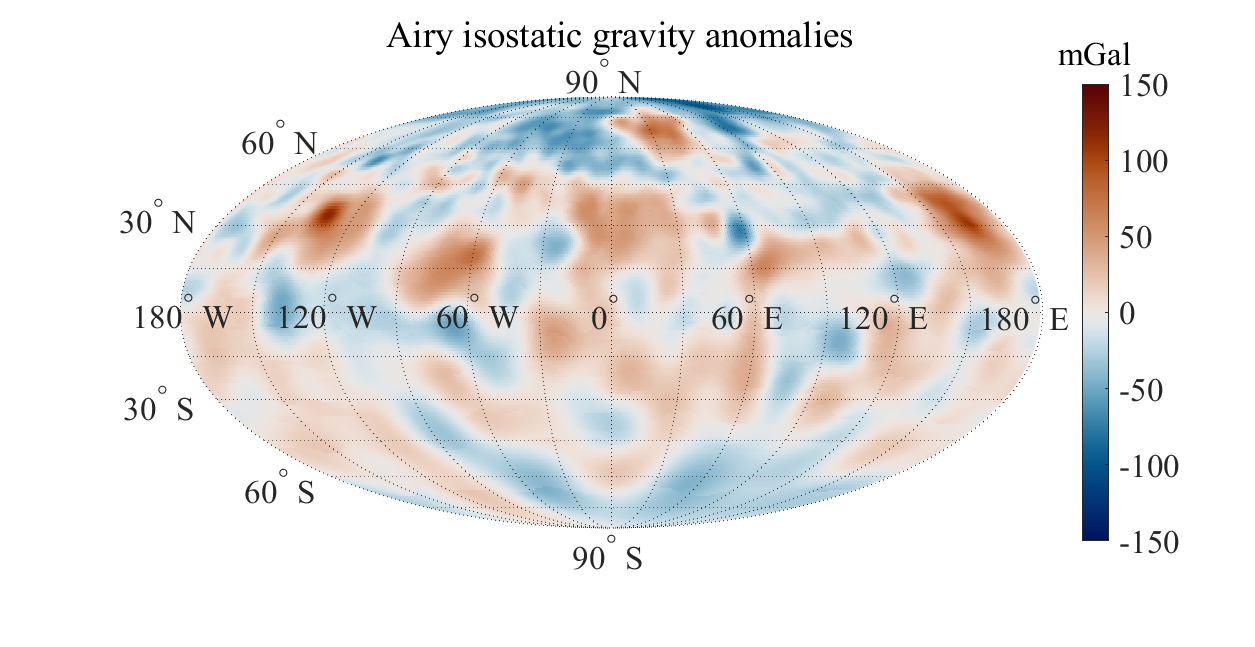
**

**Supplementary Figure** **4**. Isostatic gravity anomalies calculated using the degree strength of the MESS160A gravity field model, according to the Airy compensation model. The anomalies are computed at 50 km altitude. Maps are in Mollweide projection.

Spectral correlation between free-air and isostatic gravity anomalies

**Supplementary Figure** **5**. Spectral correlation[^7^](#Wieczorek) between free-air and isostatic anomalies considering the lithospheric deflection model (blue curve) and spectral correlation between free-air and isostatic anomalies considering the Airy model (orange curve).


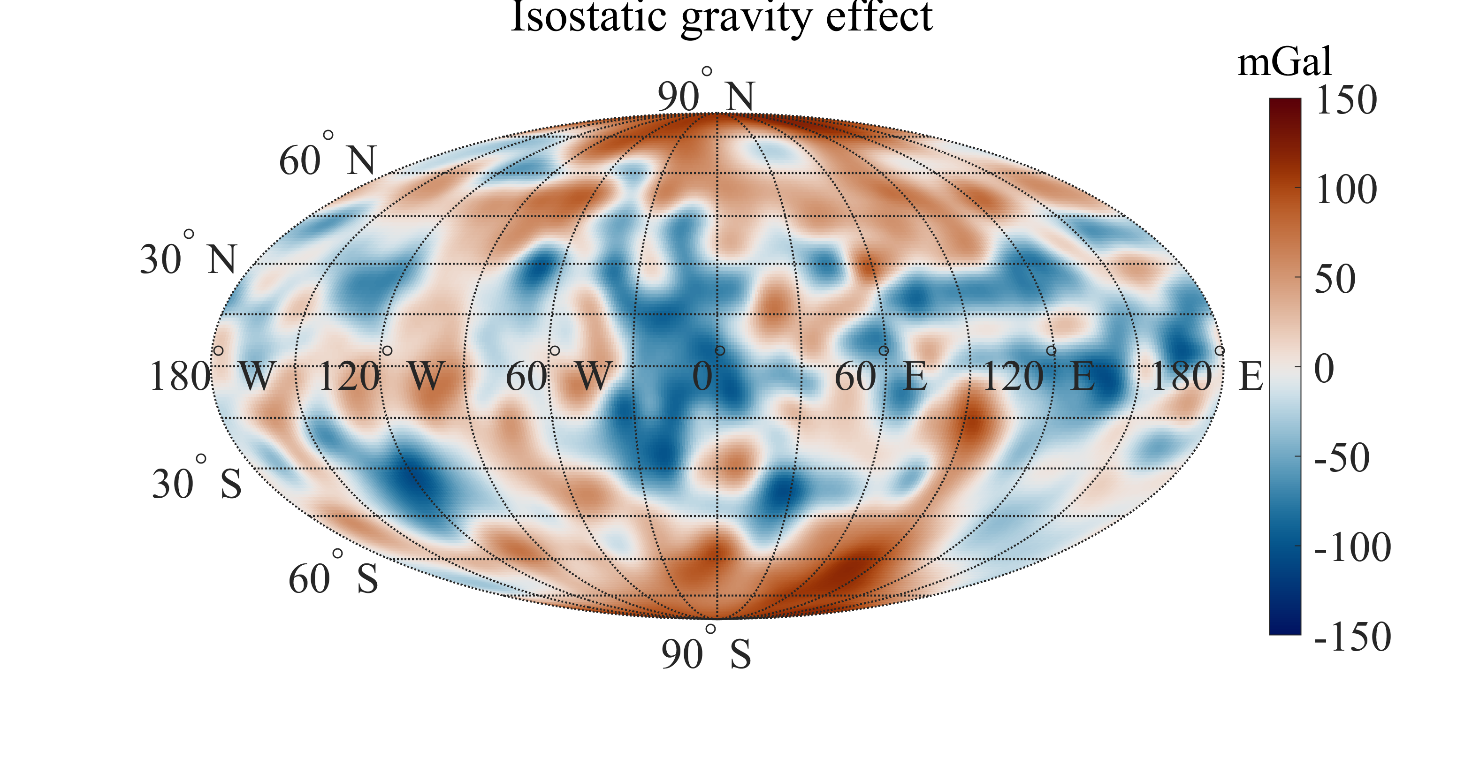


**Supplementary Figure 6.** Isostatic gravity effect calculated assuming a flexural compensation model.

**Supplementary Figure 7.** Isostatic gravity anomalies, after correcting the free-air anomalies with the topographic and isostatic gravity effects. Major features indicated are: **CM** = Caloris Montes; **CP** = Caloris Planitia; **CR** = Carneige Rupes; **DA** = Derain anomaly; **KA** = Kuiper anomaly; **NR** = Northern Rise; **NSP** = Northern Smooth Plains; **RC** = Rachmaninoff Crater; **SiP** = Sihtu Planitia; **SP** = Sobkou Planitia; **VA** = Victoria Anomaly; **CC** = Catullus Crater; **GC** = Giotto Crater. The anomalies are computed at 50 km altitude. Maps are in Mollweide projection.

**Supplementary Figure 8.** Large certain basins (solid white circles) and large suggested basins (dashed white circles)[^8^](#Fassett) associated with isostatic anomaly highs interpreted as intra-crustal magmatic intrusions.

**Supplementary Figure 9.** Large certain basins (solid white circles), large suggested basins (dashed white circles)[^8^](#Fassett) and major features identified on the topography map.


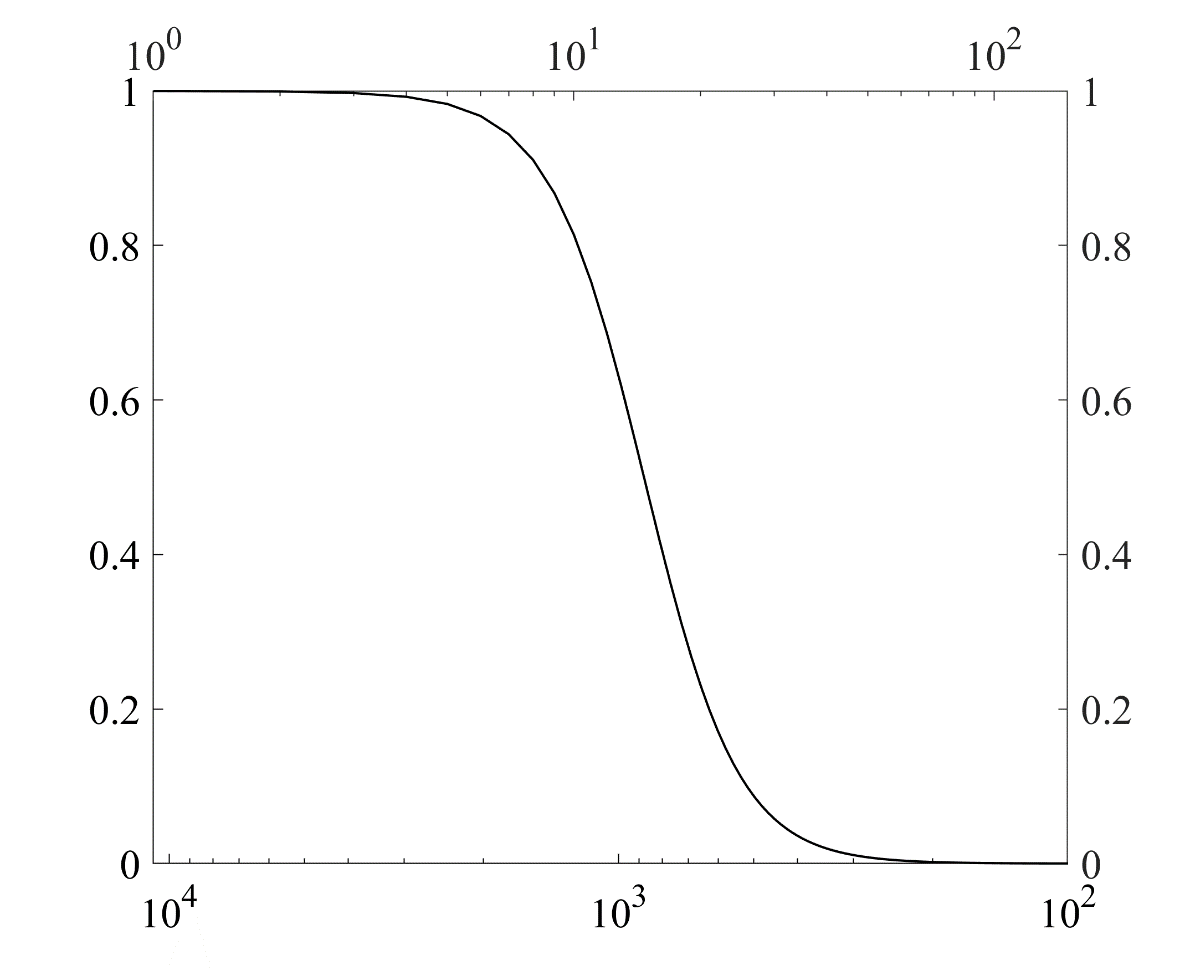


Spherical harmonic degree $l$

Wavelength $\lambda$ ($km$)

Flexural response function $\Phi(l)$

$l\gtrsim$30

$\lambda\lesssim$500 km

**Supplementary Figure 10.** Flexural response function $\Phi\left( l \right)$ calculated for subsurface loads 15 km deep, producing a density contrast of 300 kg/m^3^, and for the elastic thickness $T_{e}=$ 30 km. Intra-crustal sources, with $\lambda\lesssim$500 km, do not cause significant lithospheric flexure and their compensation roots hardly develop.

**References for Supplementary Information**

1. Genova, A. et al. Geodetic evidence that Mercury has a solid inner core. *Geophys. Res. Lett.* **46**, 3625-3633 <https://doi.org/10.1029/2018GL081135> (2019).
2. Solomon, S. C. & Anderson, B. J. The MESSENGER mission: science and implementation overview, in *Mercury: the view after MESSENGER* (eds Solomon, S. C., Nittler, L. R. & Anderson, B. J.), vol. 21, 1-29, (Cambridge Univ. Press, Cambridge, 2018).
3. Neumann, G. A. et al. Mercury shape model from laser altimetry and planetary comparisons. In *47th Lunar and Planetary Science Conference*, no. 1903, p. 2087. Retrieved from <https://www.hou.usra.edu/meetings/lpsc2016/pdf/2087.pdf> (2016).
4. Perry, M. E. et al. The low‐degree shape of Mercury. *Geophys. Res. Lett.* **42**, 6951-6958 <https://doi.org/10.1002/2015GL065101> (2015).
5. Airy, G. B. On the computations of the effect of the attraction of mountain masses. *Philosophical Transactions of the Royal Society of London* **145**, 101-104 (1855).
6. Hirt, C., Kuhn M., Featherstone, W. E. & Göttl, F. Topographic/isostatic evaluation of new-generation GOCE gravity field models. *J. Geophys. Res.* *Solid Earth* **117**, B05407, 1-16 <https://doi.org/10.1029/2011JB008878> (2012).
7. Wieczorek, M. A. Gravity and Topography of the Terrestrial Planets. *Treatise on Geophysics (Second Edition)* **10**, 153-193 <https://doi.org/10.1016/B978-0-444-53802-4.00169-X> (2015).
8. Fassett, C. I. et al. Large impact basins on Mercury: Global distribution, characteristics, and modification history from MESSENGER orbital data. *J. Geophys. Res. Planets* **117**, <https://doi.org/10.1029/2012JE004154> (2012).
